# Feasibility of Enzymatic Protein Extraction from a Dehydrated Fish Biomass Obtained from Unsorted Canned Yellowfin Tuna Side Streams: Part II

**DOI:** 10.3390/gels10040246

**Published:** 2024-04-03

**Authors:** Federica Grasso, Diego Méndez Paz, Rebeca Vázquez Sobrado, Valentina Orlandi, Federica Turrini, Lodovico Agostinis, Andrea Morandini, Marte Jenssen, Kjersti Lian, Raffaella Boggia

**Affiliations:** 1 Department of Pharmacy, University of Genova, Viale Cembrano 4, 16148 Genova, Italy; federica.grasso@edu.unige.it (F.G.); valentina.orlandi@edu.unige.it (V.O.); raffaella.boggia@unige.it (R.B.); 2 ANFACO-CECOPESCA, Department of Circular Economy, Colexio Universitario, 36310 Vigo, Spain; dmendez@anfaco.es (D.M.P.); rebeca.vazquez@anfaco.es (R.V.S.); 3 National Center for the Development of New Technologies in Agriculture (Agritech), 80121 Napoli, Italy; 4 Aimplas, Asociación de Investigación de Materiales Plásticos Y Conexas, Carrer de Gustave Eiffel, 4, 46980 Valencia, Spain; lagostinis@aimplas.es (L.A.); amorandini@aimplas.es (A.M.); 5 Nofima, Muninbakken, 9-13, 9019 Tromsø, Norway; marte.jenssen@nofima.no (M.J.); kjersti.lian@nofima.no (K.L.); 6 National Biodiversity Future Center (NBFC), 90133 Palermo, Italy

**Keywords:** marine gelatin, hydrolyzed gelatin/collagen peptides, tuna side streams, fish proteins, circular economy, rheological properties, protein hydrolysates, enzymatic extraction, zero-waste economy

## Abstract

The enzymatic extraction of proteins from fish biomasses is being widely investigated. However, little or almost no research has paid attention to the exploitation of unsorted fishery biomasses. This work is part of a larger study, Part I of which has already been published, and focuses on an extensive characterization of two collagenous samples, namely gelatin (G) and hydrolyzed gelatin peptides (HGPs), extracted from a dehydrated fish biomass coming from unsorted canned yellowfin tuna side streams. The results indicate crude protein fractions of 90–93%, pH values between 3 and 5, white–yellow colors, collagen-like FTIR spectra, and 17% in terms of total amino acid content. Viscosity and the study of dynamic viscous–elastic behavior were analyzed. Thermo-gravimetric analysis was performed to assess the residual ashes. Both samples were investigated to determine their molecular weight distribution via size-exclusion chromatography, with a higher total average molecular weight for G compared to HGPs, with values of 17,265.5 Da and 2637.5 Da, respectively. G demonstrated technological properties similar to analogous marine gelatins. HGPs demonstrated antioxidant activity as per FRAP assay. All the results open up new perspectives for the potential use of these substances in biodegradable packaging, dietary supplements, and skin care cosmetics.

## 1. Introduction

For the first time, the authors propose a scalable method represented by a single-cascade flowchart for extracting proteins from unsorted tuna side streams made up of a dehydrated mix of canned tuna leftovers obtained through an innovative industrially patented procedure [1,2]. The advantages of managing this kind of dehydrated raw material lie in the reduction in the volume of extremely perishable biomass and its increase in microbiological stability. In addition, the treatment of mixed non-separated side streams allows one to eliminate the laborious sorting step of tuna leftovers, which usually involves isolating skin and bones for collagen/gelatin extraction, representing a severe step for fish stakeholders. The exploitation of this unsorted biomass could also open up the recovery of other unsorted fish materials such as by-catches or unwanted catches [3].

The handling and valorization of unsorted biomass are considered the two main goals of the Horizon 2020—EcoeFISHent project [4] addressed to the extraction of gelatin (G) and gelatin/collagen peptides (HGPs) for the nutraceutical, cosmetic, and packaging industries.

In recent years, G and HGPs have received increasing attention for their numerous technological and functional properties, respectively. These include their gelling points, viscosity [5] and antioxidant activity [6], anti-photoaging properties [7], and anti-inflammatory and inhibitory effects on dipeptidyl peptidase-IV (DPP-IV) and angiotensin-converting enzyme (ACE) [8]. HGPs derive from enzymatic hydrolysis, which allows one to obtain low-molecular-weight peptides that show a significant increase in digestion and absorption when compared to collagen and gelatin [9].

Fish waste and fish side streams like skin, muscles, viscera, and blood have been highlighted in many research studies as sustainable sources of proteins and peptides [10]; however, little or almost no research has paid attention to the exploitation of unsorted fishery biomasses.

**Figure 1:**
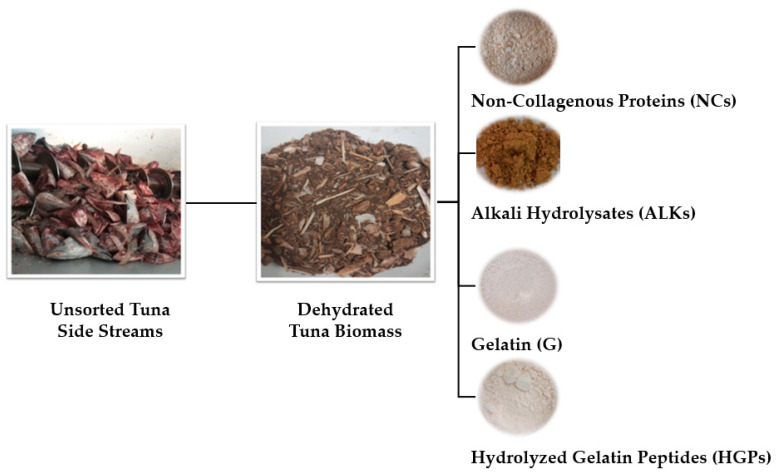
Protein fractions obtained from the proposed single-cascade flow chart extraction.

This work suggests a single-cascade flowchart for the extraction of all the protein fractions, namely gelatin (G), hydrolyzed gelatin/collagen peptides (HGPs), and non-collagenous proteins (NCs and ALKs) as reported in Figure 1.

Proposing a single-cascade extraction process minimizes, as much as possible, the process waste according to one of the principles of green extraction [11].

For G, NCs and ALKs, a preliminary characterization in terms of yield, proximate analysis, Fourier Transform Infrared Spectroscopy (FTIR) spectra, the amino acid composition, and CIELab color analysis were reported in the first paper (Part I), with a focus on oven-dried gelatin and its rheological properties (viscosity and gelling point).

Hydrolyzed gelatin peptides (HGPs) and (spray-dried) gelatin (G) are at the core of this second paper (Part II), in which the following results are discussed: proximate compositions, pH, CIELab color profiles, amino acid compositions, FTIR spectra/molecular structures, dynamic viscosity, dynamic viscous–elastic behavior (DVB), thermo-gravimetric analysis (TGA), molecular weight distributions, and antioxidant activity.

This work aims to provide a complete overview of the extracted collagenous proteins to open new perspectives of investigation for their further applications in both high-value food supplements and skin care products as well as in biodegradable and compostable packaging materials.

## 2. Results and Discussion

### 2.1. Proximate Analysis and pH Values of Extracted Gelatin and Hydrolyzed Gelatin Peptides

**Figure 2:**
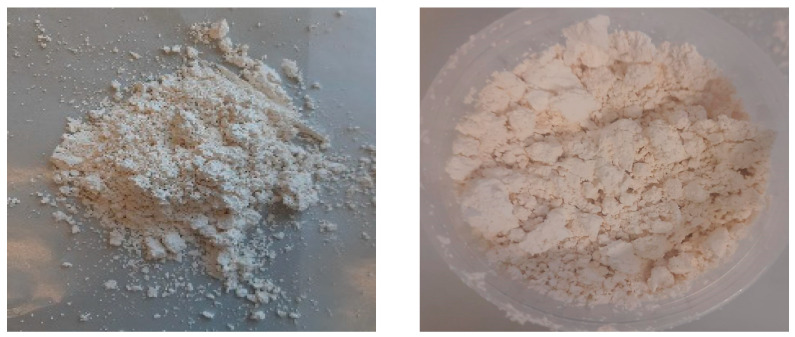
Spray-dried gelatin (G) and hydrolyzed gelatin peptides (HGPs) obtained from the proposed single-cascade flow chart extraction.

The samples of G and HGPs (Figure 2) were extracted from unsorted, mixed, and dehydrated crude tuna side streams.

The collagenous material of this biomass is mainly present in the bones and the connective tissue. Spray-drying dehydration was chosen as the most ideal drying method as it represents the most scalable process in terms of time and energy consumption. The method was compared against oven drying and lyophilizing (Figure S1). The two protein samples (of G and HGPs) were analyzed to determine their proximate compositions (residual moisture, proteins, and ashes) and pH values (Table 1). The proximate compositions of the samples were similar. In particular, the residual moisture was, in both samples, between 4 and 5% after the spray-drying dehydration treatment, which would allow one to obtain optimal and standardized results that warrant a long shelf life. The crude protein fraction of 90–93% was higher than the 82% value reported by Naiu et al. [12], referring to gelatin extracted from tuna bones, and it was in line with the result of 90%, obtained by Yang et al., for a gelatin extracted from the bones of the skipjack tuna [13]. As for HGPs, the Gelatin Manufacturers of Europe Association (GME) states that proteins make up 97% of the total weight in collagen peptides [14]. The total contents of ashes in G and HGPs were approximately 3% and 5%, respectively. One factor contributing to the increased quantity of ashes in HGPs could be explained by the addition of sodium hydroxide to the sample during the extraction process. The percentage of ashes was lower than in the ones obtained by Aisman et al., where they had between 7 and 8% in gelatin extracted from tuna bones [15]. The quantity of ashes depends on the content of minerals that are naturally present in bones. In both cases, considering the results of the proteins and low percentage of ashes, the two protein samples showed high purity, which is essential for many possible applications, such as for biomaterials, in which the high content in salt can increase the film opacity since the composition can affect the transparency [16].

Regarding the pH, it is strictly dependent on both the extraction conditions and the starting raw material. In this study, the pH was lower for G than HGPs, with the values being 3.4 and 4.8, respectively. Alfaro et al. [17] observed that the pH of the gelatin extracted from the bones of the king weakfish was around 4.

The microbiological and oxidation parameters and the presence of chemical contaminants (i.e., dioxins, PHA (benzo(a)anthracene, benzo(a)pyrene, benzo(b)fluoranthene and chrysene, metals, and PCB) were determined using the official methods and were checked on both the starting material (pre- and post-dehydration steps) and on the extracted bioactives. Some data were reported in the first part (Part I) of this research publication [1]; nevertheless, all the levels of contaminants were below the limits reported by Commission Regulation (EU) 2023/915 of 25 April 2023 on the maximum levels for certain contaminants in food.

### 2.2. Color Analysis (CIELab Color Space) of Gelatin and Hydrolyzed Gelatin Peptides

The colors of G and HGPs have been expressed as CIELab color parameters, referring to L* (lightness), a* (redness/greenness), and b* (yellowness/blueness), in Table 2. The color is considered the first aesthetic factor that defines the quality and desirability of both gelatin and gelatin peptides, even if it does not necessarily influence their functional properties. The color is mostly dependent on the dehydration performed (see Figure S1) to stabilize the product. In this study, spray-drying at 160 °C produced white-pale yellow powders for both samples, as highlighted in Figure 1. The slight differences in colors between the two powders could have been caused by the enzymatic treatment carried out to obtain HGPs, in particular on the enzyme color (dark brown), which makes HGPs’ yellowness a bit higher [18].

### 2.3. Amino Acid Analysis of Gelatin and Hydrolyzed Gelatin Peptides

**Figure 3:**
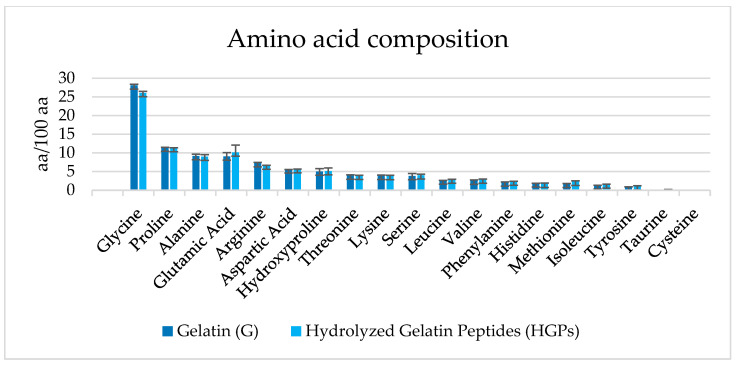
Amino acid compositions of gelatin (G) and hydrolyzed gelatin peptides (HGPs).

The amino acid compositions of G and HGPs extracted from unsorted mixed dehydrated raw tuna side streams are reported in Figure 3 and are expressed in the following format: amino acid/100 amino acids (%).

The most abundant amino acids in collagen are glycine, proline, alanine, and hydroxyproline. The glycine content was 28 and 26% for G and HGPs, respectively, while the quantities of the imino acids proline and hydroxyproline constituted, for both samples, around 16%, as was also observed by Cho et al. in gelatin extracted from skate (*Raja kenojei*) skins [19] and by Yang for gelatin hydrolysate coming from bones of the skipjack tuna (around 18%) [13]. In comparison with mammalian gelatin, the marine gelatin content in amino acids (proline and hydroxyproline) is lower, and this is the main reason why they present different physical properties [20] (viscosity, gel strength, and gelling point). Alanine and glutamic acid content represented about 10% in both samples of G and HGPs, followed by arginine at 7%.

### 2.4. Attenuated Total Reflectance Fourier Transform Infrared Spectroscopy Analysis of Gelatin and Hydrolyzed Gelatin Peptides

**Figure 4:**
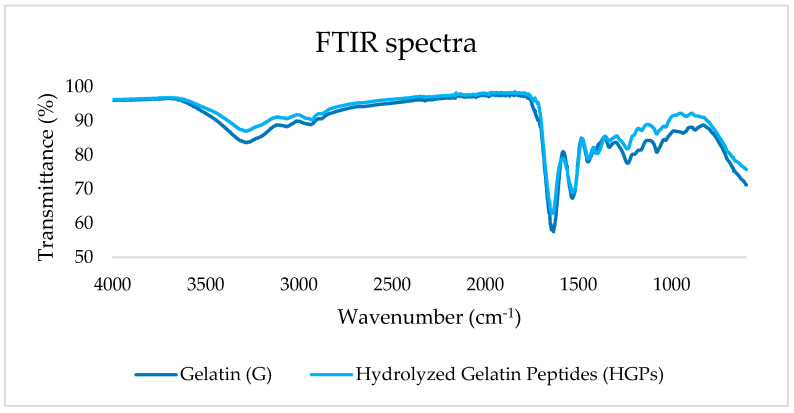
FTIR spectra of gelatin (G) and hydrolyzed gelatin peptides (HGPs).

The secondary structures of G and HGPs were evaluated through Attenuated Total Reflectance FTIR (ATR-FTIR), and the corresponding spectra are reported in Figure 4.

Collagen and gelatin have characteristic FTIR spectra absorption bands that represent amide vibrational modes (Amides A, B, I–III) and can provide information regarding the secondary structures of proteins [21]. Amide A, found at 3283/3286 cm^−1^, reflects NH stretching and, more precisely, it is believed to originate from Fermi resonance between the NH-stretching frequency and the first overtone of amide II [22]. Amide B, with low intensity peaks at 2938/2934 cm^−1^, corresponds to both NH_3_ and the asymmetric stretching vibrations of =C H. [23]. Amide I, indicative of α-helix chains [24], is represented at 1634/1644 cm^−1^ with C=O stretching [25] coupled with CN stretching, CCN deformation, and in-plane NH bending modes [26]. In collagen, this peak is found at 1655 cm^−1^ while, in the case of hydrolysis and denaturation, a shift occurs at 1645–1630 cm^−1^ due to the vibrations of proline and hydroxyproline [27]. The Amide II bands were both found at 1532 cm^−1^, associated with CN stretching and NH bending vibrations [28]. Amide III was represented by the peaks at 1237/1239 cm^−1^; the intensity of this band is related to the triple-helical structure [29]. When comparing denatured collagen to pure collagen, the IR absorption ratio 1235/1450 cm^−1^ of the latter is 1.00, and it is linked with the triple-helix integrity [30]; in this case, the ratio was equal to 0.99 for G and 0.96 for HGPs, meaning that the structure of the triple helix seemed almost totally to have been maintained in both.

### 2.5. Dynamic Viscosity of Gelatin and Hydrolyzed Gelatin Peptides

The dynamic viscosities of water solutions of G and HGPs were evaluated in both concentrations, i.e., 6.67% and 13.34%, obtaining 5.8 and 17.0 mPa·s for G and 2.1 and 3.0 mPa·s for HGPs (Table 3). This analysis is particularly important for defining possible applications of the two samples. G showed a low viscosity at the lower concentration, but the value can easily be increased considering higher concentrations unlike in HGPs, whose viscosity does not increase significantly with their concentration.

The analysis of the viscosity of protein water solutions is mostly influenced by the analytes’ temperatures and concentrations; at the same time, it also depends on not only factors that are only linked to the analyte characteristics, such as molecular weight (MW) [31] and amino acid profile, but also on ambient factors [32] like the extraction process and the pH.

Shyni et al. reported a viscosity of 4.37 mPa·s (at 6.67% and 30 °C) for gelatin extracted from skipjack tuna skin [33]. Sreeja et al. presented a value of viscosity of 4.17 mPa·s (at 6.67% and 25 °C) for *Labeo rohita* gelatin [34].

The values of HGPs in this study were similar to those of commercial hydrolyzed fish gelatines of the same molecular weight range [35,36].

### 2.6. Dynamic Viscous–Elastic Behavior of Gelatin and Hydrolyzed Gelatin Peptides

**Figure 5:**
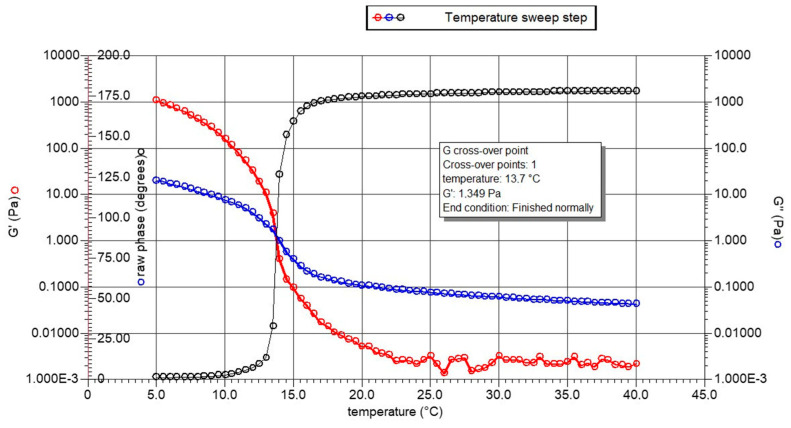
DVB, dynamic viscoelastic behavior, of gelatin (G).

The DVB is reported in Figure 5 only for G since the solution of HGPs did not produce gel in the considered temperature range as expected and reported for collagen hydrolysates in GME guidelines [14].

The gelling point of G, recorded at 13.7 °C, is represented by the temperature at which the elastic/storage modulus (G′, red curve) and the viscous/loss modulus (G″, blue curve) cross over while cooling from 40 to 5 °C.

Jeya Shakila et al. reported a gelling point equal to 16 °C for a gelatin extracted from bones of the red snapper but using a more concentrated solution of 10% [37]. Chandra et al. described a gelling point of 13.7 °C for a gelatin prepared from the swim bladders of fish Catla catla [38], performing the analysis at the same concentration that was here considered, i.e., 6.67% (*w*/*v*), and obtaining the same result.

### 2.7. Thermo-Gravimetric Analysis of Gelatin and Hydrolyzed Gelatin Peptides

**Figure 6:**
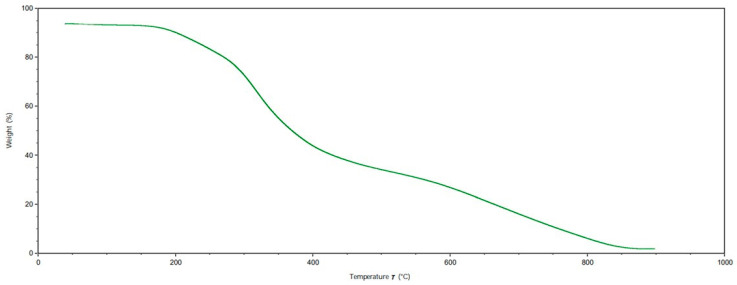
TGA (thermo-gravimetric analysis) of gelatin (G).

**Figure 7:**
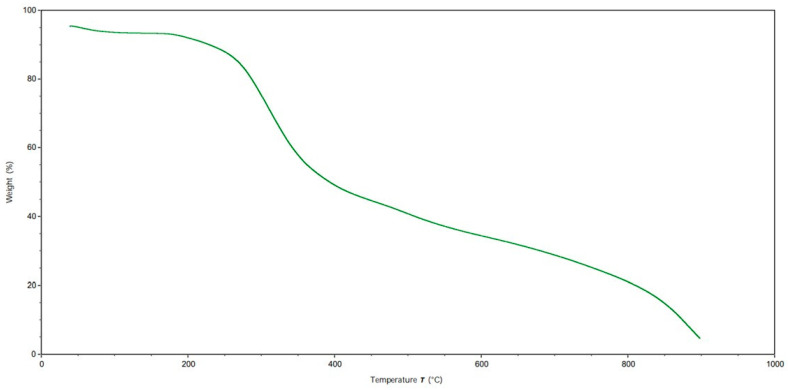
TGA (thermo-gravimetric analysis) of hydrolyzed gelatin peptides (HGPs).

The TGA showed the loss of weight of the extracted samples as a function of temperature, and it defined their thermal stability; in Figure 6 and Figure 7, the TGA results for G and HGPs are reported. After the first weight loss due to the residual humidity as adsorbed and bound water [39], the trend of weight loss followed the thermal degradation of both G and HGPs starting from 200 °C. Then, a third step of degradation was probably linked to the degradation of organic salt. From the TGA analyses, it is possible to quantify the quantity of residual ashes and to make a comparison with the incineration performed with the muffle furnace. In the case of TGA, it can be seen that G exhibited a plateau between 850 and 900 °C, which corresponded to a residual ash mass of 1.8% against the result with a muffle of 3.1%, while HGPs showed the same results in both cases (4.5% and 4.6%) but with the difference that in their cases, the plateau was not visible. It can therefore be deduced that a complete incineration of G requires higher temperatures in the muffle, but not with TGA. On the other hand, HGPs may need a temperature above 900 °C to achieve the complete incineration in both methods.

### 2.8. Determination of Molecular Weight Distribution via Size-Exclusion Chromatography of Gelatin and Hydrolyzed Gelatin Peptides

The relative size distributions of the peptides in G and HGPs were analyzed using Size-Exclusion Chromatography (SEC). As expected, the analysis showed that the G sample had a higher total average molecular weight compared to the hydrolyzed sample (HGPs), with values of 17,265.5 ± 26.2 Da and 2637.5 ± 67.2 Da, respectively. Table 4 shows the relative size distributions for the two samples, divided into categories based on size, showing that the majority of peptides in G were of 10,000 Da and above (80.2%). For the HGPs, there was a larger spread in peptide sizes, with the highest relative amount being in the range 1000–2500 Da (41.1%).

### 2.9. Antioxidant Activity of Hydrolyzed Gelatin Peptides

In the literature, it is widespread that low-molecular-weight peptides offer greater biological activities whereas high-molecular-weight peptides show greater techno-functional properties [40]. The structure of gelatin hydrolysates is made up of repetitive Gly-Pro-Ala sequences that are considered responsible for their antioxidant activity [41].

The antioxidant activity was investigated via FRAP assay and the results were expressed as ferrous equivalents (mM) using a calibration curve built with the ferrous ammonium sulphate standard solutions supplied in the kit. The utilized test measures the reducing power capacity of a compound to convert ferric iron (III) to ferrous iron (II) using a redox colorimetric reaction [42]. Different concentrations of HGPs, ranging from 25 to 200 mg/mL, were considered, corresponding to the results of 0.21 to 1.16 mM ferrous equivalents. The results can be considered higher than the one obtained, at the same conditions of temperature, by Aubry et al. [43] for collagen peptides of bovine bone (0.025–0.04 mM ferrous equivalents).

The test was also evaluated for G, obtaining the same result at 25 mg/mL, but at higher concentrations, solubility issues occurred.

## 3. Conclusions

Marine-collagen-based extracts have received significant attention both from the scientific community and from the industrial sector in the past 20 years as a “blue resource” with potential use in numerous health-related fields (food, medicine, pharmaceutics, cosmetics, and biomaterials) [44]. This work, funded by the Horizon 2020—EcoeFISHent project, aims to valorize fishing and fish industries’ side streams through the extraction of bioactives for highly valued dietary supplements and skin care products, as well as promoting their use as biodegradable and compostable biomaterials for food packaging.

In this second part (Part II) of a larger study report, a complete characterization of the two collagenous protein types, namely gelatin and hydrolyzed gelatin peptides, was reported. The single-cascade flowchart proposed turned out to represent a promising extractive process, with good prospects in terms of industrial scalability. It should be noted that starting from non-separated side streams necessarily requires pretreatment for exploiting the part richest in collagen, mainly comprising bones, from which to extract gelatin and gelatin/collagen hydrolysates. These pretreatments, especially the initial enzymatic hydrolysis, have inevitable repercussions on the quality of the gelatin that can be extracted, especially in terms of molecular weight and viscosity. However, physical properties, such as viscosity, can be released from these parameters, depending on the desired application, increasing concentration, and use of appropriate additives. Furthermore, it is important to underline that a cost–benefit analysis is crucial for the scaling up of the proposed protocol, and thus the reagents used in this research, such as the enzymatic preparation, the demineralization agent, the stabilization treatment, and so on, were chosen to be suitably scalable at the industrial scale. As far as the final drying technique was concerned, the use of a spray dryer was chosen (instead of freeze-drying) as it is more sustainable in terms of time and energy consumption. On the other hand, the cheapest oven drying technique was no longer considered, as the properties of the final products were worse.

In conclusion, given the promising results obtained, the possible uses of G and HGPs could be investigated for different applications, such as in biomaterials as regards G, which has higher molecular weight and viscosity, and in nutraceutical and cosmetic products as regards HGPs.

Furthermore, it is important to underline how, from this extraction process, it is possible to obtain other interesting products, some of which include Non-Collagenous Proteins (NCs), with a high extraction yield (about 15%) and whose properties will be further investigated in the future.

## 4. Materials and Methods

### 4.1. Samples and Chemicals

The starting raw material was made of canned raw (crude) tuna leftovers supplied by Generale Conserve (ASdoMAR^®^) company (Olbia, Italy) after a patented dehydration process performed by Themis S.p.A. that stabilizes, over time, this highly perishable biomass with a very low residual humidity. Samples of G and HGPs were extracted from raw unsorted mixed side streams of canned tuna following the abovementioned extraction protocol and spray-dried (B290 mini spray-dryer, Büchi Labortechnik AG, Flawil, Switzerland) in order to obtain dehydrated powders.

Throughout the experiments, ultrapure Milli-Q water (18 MΩ) produced by a Millipore Milli-Q system (Bedford, MA, USA) was employed. All chemicals and reagents were of analytical grade and supplied by Sigma-Aldrich Chemical Company (Steinheim, Germany) and Waters (Milford, MA, USA). The colorimetric FRAP assay kit (ab234626) was purchased from Abcam© (Abcam, Caliph, Ml, Cambridge, UK).

### 4.2. Proximate Analysis and pH of Gelatin and Hydrolyzed Gelatin Peptides

All samples were analyzed in duplicate for their characterization in terms of proximate analysis. The residual moisture was determined with official method AOAC 950.46B [45]; the protein content was estimated by Kjeldahl according to AOAC 981.10 [45] using 6.25 as conversion factor and the ash fraction was evaluated applying AOAC 942.05 [45].

The pH was recorded with a pH meter (HI5221, Hanna Instruments, Villafranca Padovana, Padova, Italy) after calibration. The measurements were carried out with solutions of 6.67% at room temperature (25 ± 1 °C).

### 4.3. Color Analysis (CIELab Color Space) of Gelatin and Hydrolyzed Gelatin Peptides

A double-beam UV-visible spectrophotometer (Agilent Cary 100 Varian Co., Santa Clara, CA, USA) filled with an integrating sphere (Varian DRA) that could uniformly disperse the light across all interior surfaces at a resolution of 1 nm was applied to measure the color in the range of 300–900 nm. Employing a reference with a white Spectralon^®^ disk, triplicate analyses were carried out. The CIE D65 illuminant was used to automatically derive the CIELab coordinates L* (lightness), a* (greenish–reddish), and b* (bluish–yellowish) from the raw spectral data using the Cary100 Color program.

### 4.4. Amino Acid Analysis of Gelatin and Hydrolyzed Gelatin Peptides

The amino acid content was chromatographically estimated after a proper protein hydrolysis procedure following Lorenzo et al.’s procedure [46] and a derivatization considering the method proposed by Domínguez et al. [47]. In particular, the chromatographic analysis of the amino acid profile was determined using an HPLC Acquity Arc (Waters Corp, Milford, MA, USA). First, 100 mg of dried material was hydrolyzed with 5 mL of hydrochloric acid 6 N for 24 h at 110 °C, then 200 mL of distilled water was added to the solution before filtration using a 0.45 μm filter. The derivatization was performed starting from 10 μL of the sample, which was buffered to pH 8.8 with AccQ-Fluor borate buffer to obtain a total volume of 100 μL. A quantity of 20 μL of AcQ-Fluor reagent (3 mg/mL in acetonitrile) was added to start the reaction of derivatization. Waters AccQ-Tag Amino Acids C18 Column (particle size of 3 µm, 3.9 mm × 150 mm) was used for the separation, setting the column heater at 37 °C and the flow rate at 1.0 mL/min. The mobile phases were prepared starting from Waters AccQ-Tag Eluent A, acetonitrile (HPLC-grade), and Milli-Q ultra-pure water as suggested by van Wandelen [48] and the injection volume was 10 µL. The detection was achieved with a Fluorescence Detector Waters 2475 with excitation length at 250 nm and emission at 395 nm.

### 4.5. Attenuated Total Reflectance Fourier Transform Infrared Spectroscopy Analysis of Gelatin and Hydrolyzed Gelatin Peptides

Through ATR-FTIR spectroscopy, a qualitative determination of samples’ molecular structures was conducted. A background reading was recorded prior to measurements. Using an FT-IR spectrophotometer (Perkin Elmer, Inc., Waltham, MA, USA), spectra were recorded at room temperature from 4000 to 600 cm^−1^ at a resolution of 4 cm^−1^, collecting 8 scans per sample.

### 4.6. Dynamic Viscosity Analysis of Gelatin and Hydrolyzed Gelatin Peptides

The dynamic viscosity of the samples was evaluated using a rotary viscometer (LV DV-II + PRO Brookfield, Brookfield Engineering, Middleboro, MA, USA) with spindle n° 18 both for single-bloom (6.67% *w*/*w*) and double-bloom (13.34% *w*/*w*) solutions (ISO 9665:1998(E)) [49] at 24 ± 1 °C with a rate of 100% rpm.

### 4.7. Dynamic Viscous–Elastic Behavior of Gelatin and Hydrolyzed Gelatin Peptides

The Dynamic Viscous–elastic Behaviors (DVBs) of the extracted samples were assessed by employing a rheometer (AR1000 Advanced Rheometer, TA Instruments, West Sussex, UK). The solutions of 6.67% (*w*/*v*) in water were made by heating them for 30 min at 60 °C and letting them cool to room temperature [50].

Using a 2° Al plate geometry of 40 mm diameter and a temperature ramp of 0.5 °C/min, the gelling point was measured throughout a range of 5–40 °C. At a frequency of 1 Hz, an oscillating stress of 3.0 Pa was applied at a 500 μm gap. Temperature was used to monitor the elastic modulus (G′) and viscous modulus (G″) in accordance with the findings given by Gómez-Guillén et al. [51]. The gel point coincided with the cross-over point between the modules (G′ and G″) during the cooling process (from 40 to 5 °C).

### 4.8. Thermo-Gravimetric Analysis of Gelatin and Hydrolyzed Gelatin Peptides

The thermal properties of gelatin (G) and hydrolyzed gelatin peptides (HGPs) were studied using a thermo-gravimetric analyzer (TA Instruments TGA QIR5000, New Castle, DE, USA) as per method UNE-EN ISO 53375-3 [52] following the temperature program of first heating cycle from 30 to 900 °C at a rate of 10.00 °C/min under 20 mL/min N_2_ flux and a repetition in air.

### 4.9. Molecular Weight Distributions and Size-Exclusion Chromatography of Gelatin and Hydrolyzed Gelatin Peptides

The molecular weight distribution of the peptides was determined using SEC. The method had been described previously by Wubshet et al. [53] and was conducted with minor differences. The dried protein samples, i.e., of HGPs and G, were rehydrated to a final concentration of 20 mg/mL prior to injection. The analysis was performed on a Prominence Shimadzu HPLC system (Shimadzu, Nishinokyo Kuwabara-cho, Nakagyo-ku, Japan), coupled to a Prominence diode array detector (Shimadzu), using a BioSep-SEC-s2000 column (300 × 7.8 mm) (Phenomenex, Værløse, Denmark). The PDA detection was set to 214 nm, with 640 ms intervals. The injection volume was 10 μL, and each sample was injected twice. The separation was performed at 30 °C. Isocratic elution was carried out (mobile phase: 30% acetonitrile, with 0.05% trifluoroacetic acid), with a flow rate of 0.9 mL/min for 17 min. After the sample separation, the column was washed for three minutes using 0.10 M NaH_2_PO_4_, before the column was re-calibrated for 25 min using the aforementioned mobile phase. The chromatographic runs were controlled using the version 5.86 of LabSolutions™ CL Realtime Analysis software (Shimadzu). Molecular weight calculations were performed in the elution period lasting from 5 to 17 min, in which the peptides were eluting. The parallels were compared using Pearson correlations based on retention time and intensity as measured by the PDA at 214 nm. The molecular weight calculations were performed in version 2402 of Microsoft Excel (Microsoft Corporation, Seattle, WA, USA) using a calibration curve constructed with molecular weight standards. The standards with known molecular weights used for calibration were as follows: Carbonic anhydrase, Lysozyme, Cytochrome c from bovine heart, Aprotinin from bovine lung, Insulin chain B oxidized from bovine pancreas, Renin substrate tridecapeptide porcine, Angiotensin II human, Bradykinin fragment 1–7, [D-Ala2]-Leucine encephalin, Val-Tyr-Val, and L-Tryptophane. The standards ranged from 30 kDa to 0.204 kDa.

### 4.10. Antioxidant Activity of Hydrolyzed Gelatin Peptides as Per FRAP Assay

The total antioxidant activity was spectrophotometrically assessed using a commercial assay kit called FRAP following the manufacturer’s instructions [54]. In this method, the antioxidant power is determined through the reduction of ferric iron (Fe III) to ferrous iron (Fe II), which generates a blue color with a maximum value of absorption at 594 nm. The reaction was carried out in flat-bottom polystyrene 96-well plates using a microplate reader for the measurements. Samples solutions were prepared in water at concentrations ranging from 25 to 200 mg/mL and ferrous ammonium sulphate standard curve with R^2^ = 0.998 was used to calculate the antioxidant capacity, reporting the results as Fe II equivalents (mM).

### 4.11. Statistical Analysis

All analyses were performed at least in duplicate, and results were expressed as the average mean values ± standard errors. The Excel Data Analysis Tool (Microsoft Corporation, Seattle, WA, USA) was used to examine the data. Analysis of variance (ANOVA) was applied to examine the mean significant differences between the samples at a significance level of 0.05.

## Figures and Tables

**Table 1:** Proximate analysis and pH of gelatin (G) and hydrolyzed gelatin peptides (HGPs).

	Gelatin (G)	Hydrolyzed Gelatin Peptides (HGPs)
Residual moisture ^1^ (g/100 g)	4.6 ± 0.3	4.1 ± 0.1
Proteins ^1^ (g/100 g)	92.9 ± 0.8	90.0 ± 0.7
Ashes ^1^ (g/100 g)	3.1 ± 0.6	4.6 ± 0.2
pH ^1^	3.4 ± 0.1	4.8 ± 0.1

^1^ Results are expressed as means ± standard errors (*n* = 2).

**Table 2:** Color parameter of gelatin (G) and hydrolyzed gelatin peptides (HGPs).

	Gelatin (G)	Hydrolyzed Gelatin Peptides (HGPs)
CIELab ^1^	L* = 88.66 ± 0.01 a* = 0.41 ± 0.01 b* = 5.89 ± 0.01	L* = 88.62 ± 0.01 a* = 0.46 ± 0.01 b* = 7.71 ± 0.01

^1^ Results are expressed as means ± standard errors (*n* = 3).

**Table 3:** Dynamic viscosity of gelatin (G) and hydrolyzed gelatin peptides (HGPs).

Dynamic Viscosity (mPa·s)	Gelatin (G)	Hydrolyzed Gelatin Peptides (HGPs)
6.67% (*w*/*w*) ^1^	5.8 ± 0.4	2.1 ± 0.1
13.34% (*w*/*w*) ^1^	17.0 ± 1.0	3.0 ± 0.3

^1^ Results are expressed as means ± standard errors (*n* = 3).

**Table 4:** Molecular weight distributions of gelatin (G) and hydrolyzed gelatin peptides (HGPs).

Molecular weight ranges (Da) ^1^	G (relative amount %) ^1^
20,000+	40.4 ± 1.6
10,000–20,000	39.8 ± 2.3
5000–10,000	8.7 ± 0.1
0–5000	11.2 ± 0.6
**Molecular weight ranges (Da)**	**HGPs (relative amount %)**
4000+	21.0 ± 1.1
2500–4000	15.7 ± 0.1
1000–2500	41.1 ± 0.5
0–1000	22.4 ± 0.5

^1^ Results are expressed as means ± standard errors (*n* = 2).

## Data Availability

Data are contained within the article.

## Supplementary Materials

The following supporting information can be downloaded at: https://www.mdpi.com/article/10.3390/gels10040246/s1, Figure S1. Freeze-dried Gelatin and Oven-dried Gelatin.
