# Supplementary figures and images for: Feasibility of Enzymatic Protein Extraction from a Dehydrated Fish Biomass Obtained from Unsorted Canned Yellowfin Tuna Side Streams: Part II
Source: Gels. 2024 Apr 3;10(4):246. doi: 10.3390/gels10040246 (PMC11049478; doi:10.3390/gels10040246)

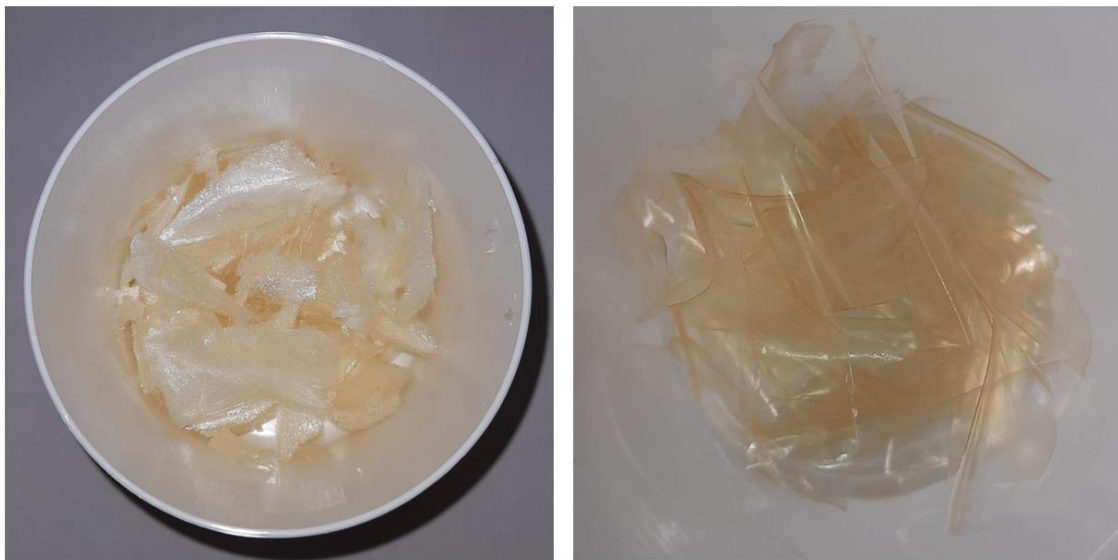

**Figure S1.** Freeze-dried Gelatin and Oven-dried Gelatin.

Supplement: Supplementary file 1 [file gels-10-00246-s001.zip › gels-2913861-supplementary.pdf]
